# The semi-quantitative cardiac arrest brain ischemia (CABI) score for magnetic resonance imaging predicts functional outcome after cardiac arrest

**DOI:** 10.1186/s13054-025-05595-1

**Published:** 2025-08-20

**Authors:** Isabelle Arctaedius, Johan Wassélius, Margareta Lang, Mattias Drake, Mikael Johnsson, Hans Friberg, Christoph Leithner, Martin Kenda, Anna Lybeck, Marion Moseby-Knappe

**Affiliations:** 1 Department of Clinical Sciences, Anaesthesia & Intensive Care, Lund University, Skåne University Hospital, Entrégatan 7, Lund, 222 42 Sweden; 2 Department of Clinical Sciences, Medical Imaging and Physiology, Lund University, Skåne University Hospital, Lund, Sweden; 3 Department of Clinical Sciences, Radiology, Lund University, Helsingborg Hospital, Helsingborg, Sweden; 4 Department of Radiology, Helsingborg Hospital, Helsingborg, Sweden; 5 Department of Clinical Sciences, Anaesthesia & Intensive Care, Lund University, Skåne University Hospital, Malmö, Sweden; 6 Department of Neurology and Experimental Neurology, Charité– Universitätsmedizin Berlin, Corporate member of Freie, Universität Berlin and Humboldt-Universität zu Berlin, Berlin, Germany; 7 Department of Clinical Sciences, Neurology and Rehabilitation Medicine, Lund University, Skåne University Hospital, Lund, Sweden

**Keywords:** Cardiac arrest, Prognostication, MRI, Neuroimaging

## Abstract

**Background:**

Magnetic resonance imaging (MRI) is recommended by guidelines to evaluate the severity of brain ischemia after cardiac arrest for functional outcome prediction. However, standardized assessment criteria are lacking. We have developed a semi-quantitative Cardiac Arrest Brain Ischemia (CABI) score to assess the extension of brain ischemia on MRI.

**Objectives:**

To evaluate the prognostic performance of a novel semi-quantitative CABI score in comparison to qualitative routine radiological MRI assessment after cardiac arrest.

**Methods:**

A retrospective, multicenter observational study of adults (≥ 18 years-old) admitted to intensive care after cardiac arrest at four Swedish hospitals from 2014 to 2018.

Four radiologists, blinded to clinical information except patient age, assessed the MRI as per guideline recommendations and according to the CABI score. The CABI score evaluates extension of ischemia in 12 predefined territories (3 cortical and 2 deep grey nuclei territories bilaterally based on vascular supply; the cerebellum; and the brainstem) from 0–3 (0 = No acute ischemic lesion; 3 ≥ 50% of the territory). The CABI score ranges from 0–36. Poor functional outcome was defined as Cerebral Performance Category 3–5 assessed at 2–6 months post-arrest. Prognostic ability was evaluated with sensitivity and specificity and with the area under the receiver operating characteristics curve (AUC). Inter-rater reliability was calculated by Fleiss´ Kappa or intraclass correlation coefficient.

**Results:**

Ninety (n = 90) patients examined with MRI at median 5 days (IQR: 3.7−6.3) were included Median age was 63.7 years (IQR: 58.3−70.5), 74.4% (n = 67) were men and 85.6% (n = 77) had a poor functional outcome. Among four blinded raters, routine qualitative assessment according to guidelines showed 84.6–100% specificity and 61–76.6% sensitivity for poor outcome. Inter-rater reliability for the qualitative assessment was moderate (Fleiss κ = 0.64, 95% CI: 0.53−0.75). The CABI score achieved an AUC of 0.87–0.92 (p< 0.05 vs. routine assessment) and a sensitivity of 59.7−85.7% while maintaining high specificity (76.9–100%) at cut-off ≥ 8 points. The intraclass correlation coefficient (ICC) for the CABI score was 0.67 (95% CI: 0.58–0.75), reflecting moderate agreement among raters.

**Conclusion:**

A semi-quantitative approach to MRI evaluations after cardiac arrest may increase the prognostic accuracy compared to non-standardized routine assessment.

**Trial registration:**

SweCrit biobank, retrospectively registered at ClinicalTrials.gov no. NCT04974775 18th of June 2021.

**Supplementary Information:**

The online version contains supplementary material available at 10.1186/s13054-025-05595-1.

## Background

The European Resuscitation Council (ERC) and the European Society of Intensive Care Medicine (ESICM) recommend evaluating the presence of diffuse and extensive anoxic injury on brain CT or magnetic resonance imaging (MRI) to predict poor functional outcome after cardiac arrest [1–3]. The level of evidence is low and standardized criteria are lacking. Similarly, the Neurocritical Care Society (NCS) guidelines advise using ‘a diffuse pattern of restricted diffusion’ across vascular territories bilaterally in both the anterior and posterior circulation, involving the cerebral cortex and deep grey matter, for prognostication [1–3]. The International Liaison Committee on Resuscitation (ILCOR) suggests using the absence of diffusion restriction on MRI, performed between 3 and 7 days post-arrest, to predict good functional outcomes [4].

Acute hypoxic-ischemic brain injury can be assessed on MRI using diffusion-weighted imaging (DWI) where acute ischemia is characterized by restricted diffusion or on T2-weighted sequences such as fluid-attenuated inversion recovery (FLAIR) [5–11]. A recent study showed high sensitivity and specificity for prediction of poor outcome based on a lesion pattern score on DWI [12, 13]. However, this score requires manual placement of regions of interest (ROIs) in the cerebral cortex, the cerebellar cortex, the hippocampi, the basal ganglia, the thalami and the brainstem, which may limit practicality in clinical routine care.

Several semi-qualitative scoring tools for evaluating DWI and FLAIR have been developed [5, 6, 8, 9]. In general, higher scores, particularly in cortical regions and in deep grey nuclei, were associated with poor outcome post-arrest and all tools demonstrated moderate-high inter-rater reliability. Recently, a scoring system for DWI modified from the Alberta Stroke Program Early CT Score (ASPECTs) demonstrated high prognostic performance [10]. The ASPECT score is widely used to assess brain ischemia on CT and guide treatment decisions regarding thrombolysis and thrombectomy [14, 15]. However, ASPECTs only assess the middle cerebral artery (MCA) territory, although adaptations have been developed for the posterior circulation [14–16].

Diffusion tensor imaging (DTI) is another MRI sequence which has been shown to have high accuracy in predicting clinical outcome when performed within one week of arrest [11]. However, DTI is not commonly used in clinical routine care.

We have developed a semi-quantitative Cardiac Arrest Brain Ischemia (CABI) score. The aim of the current study is to compare the prognostic performance of this score with the qualitative assessment recommended by the ERC/ESICM guidelines for prediction of poor functional outcome after cardiac arrest.

## Methods

### Study setting and patients

This was a retrospective observational multicenter study of post-cardiac arrest in adult patients (≥ 18 years of age) included between 2014 and 2018 in the SweCrit biobank (ClinicalTrials.gov, no. NCT04974775) at four Intensive Care Units in southern Sweden, after in- or out-of-hospital cardiac arrest [17]. Results are presented according to the Standards for Reporting Diagnostic accuracy studies (STARD).

International Classification of Diseases, 10th Revision (I46.0) in the intensive care registry was used to identify cardiac arrest patients. Post-cardiac arrest care including neuroprognostication was provided according to current ERC/ESICM guidelines [18]. MRI was performed on clinical indication at discretion of the treating physician [17].

### Data sources

Medical records, the International Cardiac Arrest Registry, the local intensive care registry, and the Swedish population registry were used to collect patient data.

### Ethical approval and informed consent

The study protocol was approved by the Regional Ethical Review Board in Lund, Sweden (2014/47 and 2015/267) and the Swedish Ethical Review Authority (2022-02681-01). Informed consent was obtained from patients who regained mental capacity.

### MRI evaluation

Four radiologists with 6–18 years neuroradiology experience independently assessed all brain MRI examinations blinded to clinical information except for patient age. Two raters were neuroradiology consultants and two were general radiologists with a special interest and expertise in neuroradiology.

**Fig. 1:**
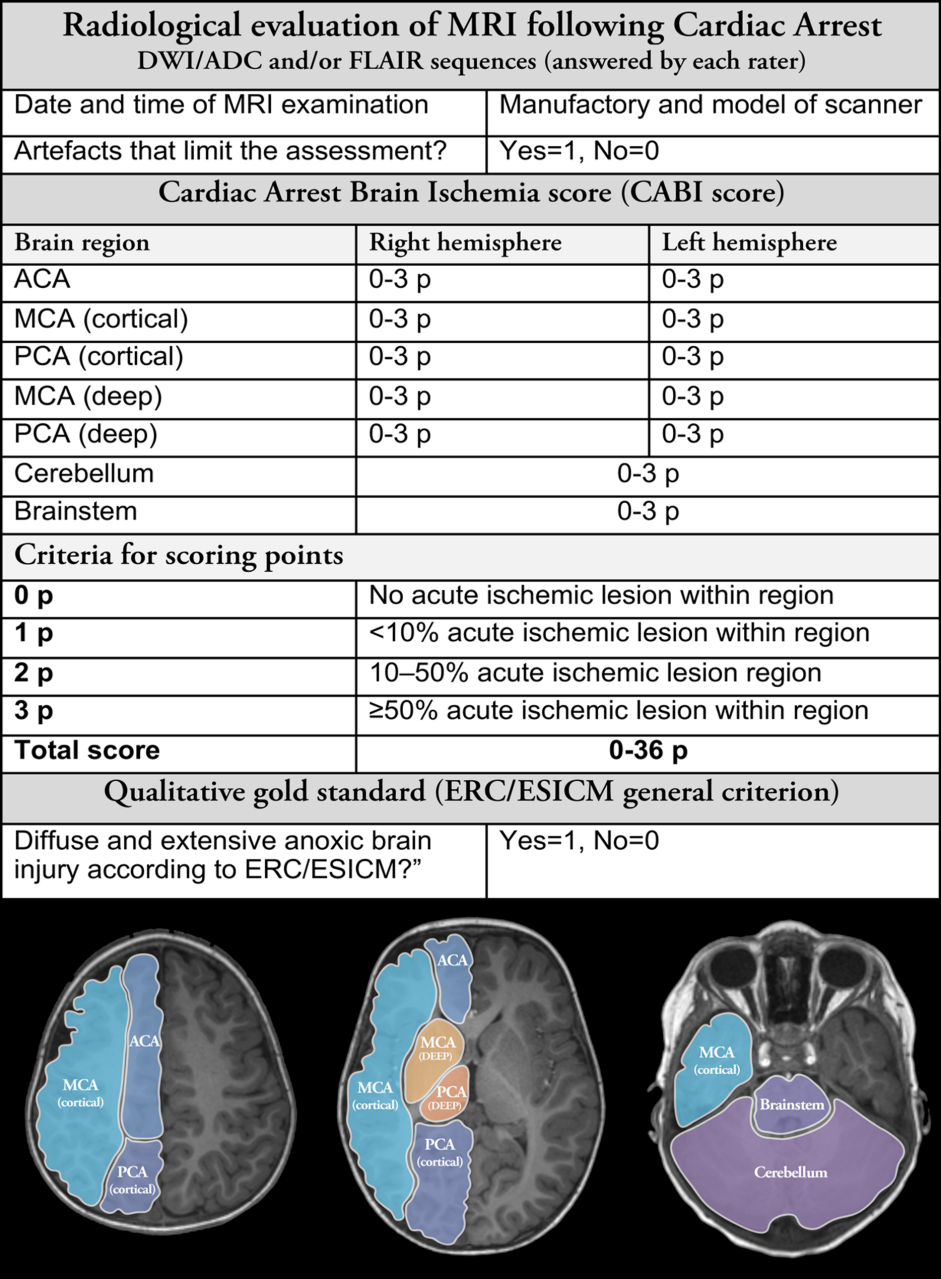
The radiological checklist for brain MRI evaluation. The figure shows the checklist for brain MRI evaluation used by the four raters. The checklist includes the semi-quantitative Cardiac Arrest Brain Ischemia (CABI) score and the qualitative assessment as per ERC/ESICM recommendations. The CABI score is based on vascular/anatomical regions and has maximum of 36 points: anterior cerebral artery, middle cerebral artery (cortical respectively deep), posterior cerebral artery (cortical respectively deep), cerebellum and the brainstem.*DWI; Diffusion-Weighted Imaging*,* ERC; European Resuscitation Council*,* ESICM; European Society of Intensive Care Medicine*,* FLAIR; Fluid-Attenuated Inversion Recovery*,* MRI; Magnetic Resonance Imaging*

All radiologists were instructed to rate chemical hits with a combined assessment including DWI, ADC and FLAIR sequences. Both according to the general criterion of diffuse and extensive restriction according to current ERC/ESICM recommendations and the CABI score (Fig. 1) [1]. One rater (Rater 1) rated all examinations according to the general criterion twice with 5 months in between to evaluate intra-rater variability.

### The CABI score

The CABI score (Fig. 1) is based on evaluation of ischemic lesions in 12 brain regions, 3 cortical and 2 deep grey nuclei regions based on the main vascular supply (the ACA-, MCA- and PCA-territories) in both hemispheres separately; the cerebellum and the brainstem.

Each of the 12 regions are assigned 0–3 points depending on the extension of ischemic lesions (0: No acute ischemic lesion; 1: Acute ischemic lesion in < 10% of the region; 2: Acute ischemic lesion in 10–50% of the region; and 3: Acute ischemic lesion in ≥50% of the region).

Cortical and deep grey nuclei regions were scored for left and right hemisphere lesions separately, the cerebellum and brainstem were scored once per patient (Fig. 1). The CABI score consequently ranges from 0 (no ischemic lesions) to maximum 36 points.

Additionally, we performed a predefined sensitivity analysis where we excluded deep grey matter (MCA and PCA deep in Fig. 1). The maximum score in the sensitivity analysis was 24 points.

### Clinical outcome assessment

Functional outcome was assessed at any type of healthcare follow-up appointment at 2–6 months post-arrest using the Cerebral Performance Category Scale (CPC), dichotomized to good outcome (CPC 1–2: normal performance to moderate disability) or poor outcome (CPC 3–5: severe cerebral disability, coma, and brain death). Reasons for withdrawal of life-sustaining treatment were classified as one or more of the following: neurologic, circulatory failure/multiple organ failure, comorbidity, and ethical reasons. Withdrawal of life-sustaining therapies was based on the current guidelines for post-resuscitation care [18].

### Statistics

Descriptive data are presented as median [interquartile range] for continuous variables and as n (%) for categorical variables.

Sensitivity, specificity, positive predictive value (PPV) and negative predictive value (NPV) and area under the receiver operating characteristics curve (AUC) presented with 95% confidence interval (CI) was used for the radiological assessments. Difference in AUC was tested using DeLong’s method. A p-value of < 0.05 was interpreted as statistically significant.

Inter-rater reliability was calculated by Fleiss´ Kappa for binary data and intraclass correlation coefficient (ICC) for ordinal data. Cohen´s Kappa was used to calculate intra-rater reliability for binary data. The coherence between the qualitative radiological assessment and the CABI score was calculated in numbers and percentages. All statistical analysis was performed using R Studio Version 2024.09.0 + 375.

## Results

### Study population

**Fig. 2:**
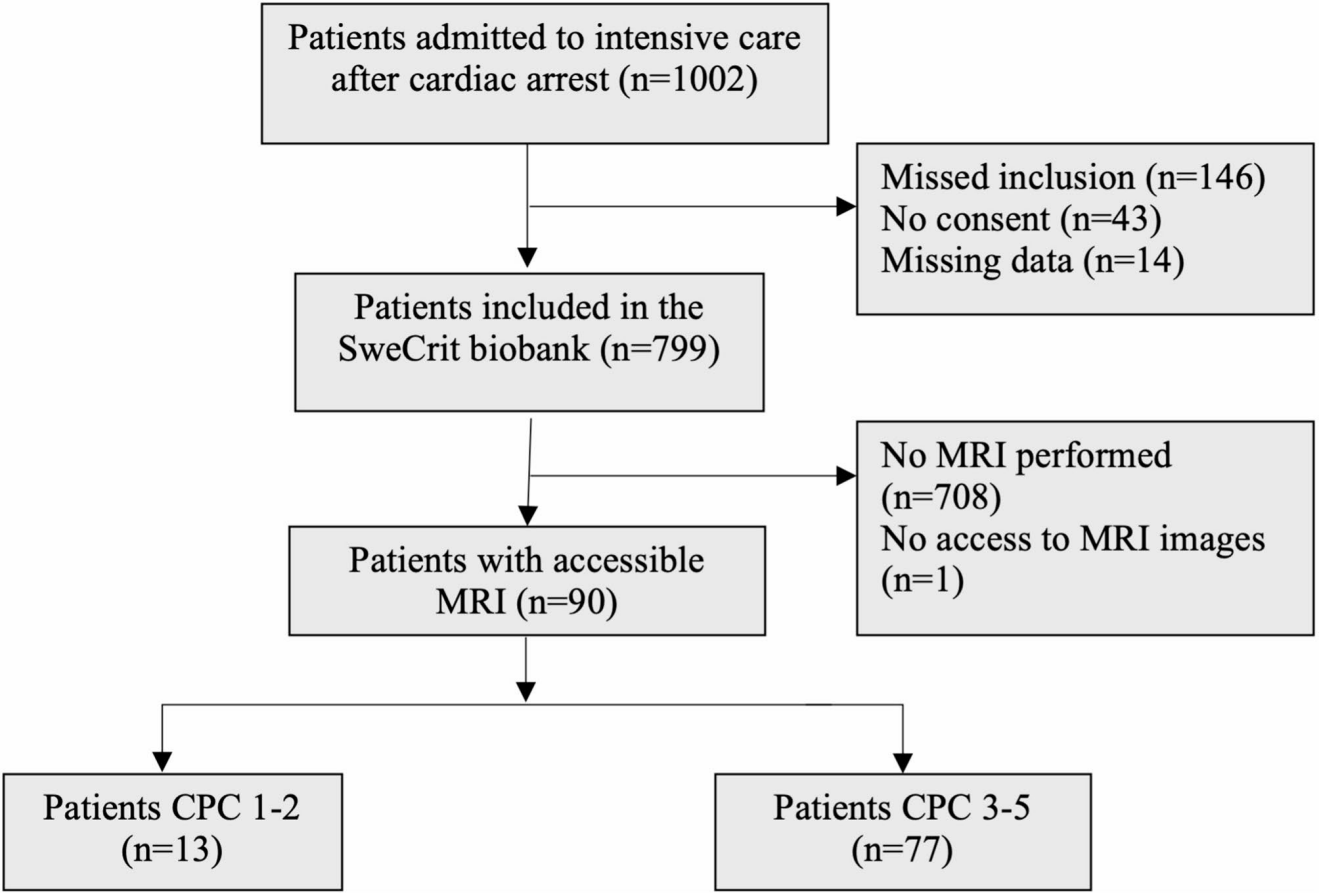
Flowchart of patient inclusion. Flowchart showing included and excluded patients in the cohort. Functional outcomes at 2-6 months assessed according to the Cerebral Performance Category (CPC) scale, dichotomised into good (CPC 1–2) and poor (CPC 3–5). *CPC; Cerebral Performance Category*,* MRI; Magnetic Resonance Imaging*

Screening of the SweCrit biobank from 2014 to 2018 resulted in 799 patients of which 91 were examined with MRI post arrest. Of these 91 patients, 1 was excluded due to unavailability of MRI images, resulting in a final study population of 90 patients (Fig. 2).

Patient characteristics of the included (*n* = 90) and the excluded patients (*n* = 709) are presented in Table 1. Median age of included patients was 63.7 years (IQR 58.3–70.5), of which 74 patients (82%) had an out-of-hospital cardiac arrest. Median time from cardiac arrest to brain MRI was 5 days (IQR 3.7–6.3). The majority of MRI scans were performed with Siemens or GE scanners (Table 1). Functional outcome was poor in 77 (86%) patients. Withdrawal-of-life-sustaining therapies was performed in 63 patients (70%) with a median time of 5.9 days (IQR 4.2–7.2) after cardiac arrest. Perceived poor neurological prognosis was the most prevalent reason (61/70, 97%) to withdraw life-sustaining therapy.

**Table 1:** Patient characteristics

Variable	Included (*n* = 90)	Excluded (*n* = 709)
Age	63.7 [58.3–70.5]	70.4 [61.4–77.4]
Sex, Male	67 (74.4)	489 (69)
**Medical history**		
Myocardial infarction	9 (10)	122 (17.2)
Congestive heart failure	6 (6.7)	144 (20.3)
Hypertension	38 (42.2)	278 (39.2)
Liver disease	2 (2.2)	22 (3.1)
Renal disease	6 (6.7)	89 (12.6)
Diabetes	25 (27.8)	171 (24.1)
Cerebrovascular disease	9 (10)	66 (9.3)
Chronic obstructive pulmonary disease	8 (8.9)	89 (12.6)
Dementia/cognitive impairment	1 (1.1)	40 (5.6)
Solid tumor	12 (13.3)	88 (12.4)
**Cardiac arrest variables**		
Out-of-hospital cardiac arrest	74 (82.2)	480 (57.5)
Minutes to return of spontaneous circulation	20 [13-32.2]	20 [10–35]^f^
Witnessed cardiac arrest	68 (75.6)	563 (79.4)^c^
Shockable rhythm	36 (40)^f^	282 (39.8) ^i^
Cardiac cause of arrest^a^	51 (56.7)^c^	426 (60.1) ^e^
**Admission data**		
Glasgow coma scale motor response	1 [1–1]^d^	1 [1–4]^h^
Circulatory shock	23 (25.6)	258 (36.4) ^d^
**Intensive care data**		
Patients regaining consciousness	22 (24.4)^c^	363 (51.2) ^f^
Days from cardiac arrest to awakening	3 [1–6]	1 [0–2]^c^
Neuron-specific enolase 48 h after cardiac arrest	45 [26.5–106]	47.5 [21–147]
**Magnetic resonance imaging data**		
Days to MRI	5 [3.7–6.3] (*n* = 90)	2.8 [2.8–2.8] (*n* = 1)
**Magnetic resonance imaging scanners**		
Siemens	51 (56.7)	–
GE	33 (36.7)	–
Philips	2 (2.2)	–
Other/unknown	4 (4.4)	–
**Withdrawal of life-sustaining therapies (WLST)**		
WLST^b^	63 (70)	272 (38.4)
WLST due to poor neurological prognosis	61 (96.8)	202 (74.3) ^d^
Days from cardiac arrest to WLST	5.9 [4.2–7.2]	2.7 [0.9-4]
**Follow-up**		
Good outcome, CPC 1–2	13 (14.4)	260 (36.7) ^g^
Poor outcome, CPC 3–5	77 (85.6)	444 (62.6) ^g^

Continuous variables are presented as median [interquartile range] and categorical variables as n (%). Proportions (%) are within the groups

^a^ Retrospectively diagnosed during the hospital stay

^b^ Multiple reasons for WLST possible including neurological, circulatory, multi-organ failure, comorbidities, or ethical reasons

Missing: ^c^*n*=1 ^d^*n*=2 ^e^*n*=3 ^f^*n*=4 ^g^*n*=5 ^h^*n*=31 ^i^*n*= 33

*CPC Cerebral*

*Performance Category*,* WLST withdrawal of life-sustaining therapy*

### Qualitative assessment according to ERC/ESICM recommendations

Evaluating the general criterion of presence of diffuse and extensive diffusion restriction on brain MRI according to ERC/ESCIM recommendations had a specificity of 84.6–100% (range of all four raters) and a 61–76.6% sensitivity for prediction of poor outcome (Table 2.).

**Table 2:** Specificity, sensitivity, positive predictive value and negative predictive value of qualitative and semi-quantitative MRI assessment

**Qualitative assessment per ERC/ESICM recommendations**									
	Rater	Specificity % (95%CI)	Sensitivity % (95%CI)	PPV % (95%CI)	NPV % (95%CI)	TP	FP	TN	FN
	1	92.3 (66.7-98.6)	76.6 (66-84.7)	98.3 (91.1-99.7)	40 (24.6-57.7)	59	1	12	18
	2	84.6 (57.8-95.7)	76.6 (66-84.7)	96.7 (88.8-99.1)	37.9 (22.7-56)	59	2	11	18
	3	100 (77.2-100)	61 (49.9-71.2)	100 (92.4-100)	30.2 (18.6-45.1)	47	0	13	30
	4	100 (77.2-100)	61 (49.9-71.2)	100 (92.4-100)	30.2 (18.6-45.1)	47	0	13	30

**Semi-quantitative CABI score**									
Score (0-36p)	Rater	Specificity % (95%CI)	Sensitivity % (95%CI)	PPV % (95%CI)	NPV % (95%CI)	TP	FP	TN	FN
≥4	**1**	84.6 (57.8-95.7)	83.1 (73.2-89.9)	97 (89.6-99.2)	45.8 (27.9-64.9)	64	2	11	13
	**2**	46.2 (23.2-70.9)	98.7 (93-99.8)	91.6 (83.6-95.9)	85.7 (48.7-97.4)	76	7	6	1
	**3**	84.6 (57.8-95.7)	80.5 (70.3-87.8)	95.7 (89.3-99.1)	42.3 (25.5-61.1)	62	2	11	15
	**4**	100 (77.2-100)	77.9 (67.5-85.7)	100 (94-100)	43.3 (27.4-60.8)	60	0	13	17
≥5	**1**	84.6 (57.8-95.7)	81.8 (71.8-88.8)	97 (89.5-99.2)	44 (26.7-62.9)	63	2	11	14
	**2**	53.8 (29.1-76.8)	97.4 (91-99.3)	92.6 (84.8-96.6)	77.8 (45.3-93.7)	75	6	7	2
	**3**	100 (77.2-100)	76.6 (66-84.7)	100 (93.9-100)	41.9 (26.4-59.2)	59	0	13	18
	**4**	100 (77.2-100)	71.4 (60.5-80.3)	100 (93.5-100)	37.1 (23.2-53.7)	55	0	13	22
≥8	**1**	100 (77.2-100)	77.9 (67.5-85.7)	100 (94-100)	43.3 (27.4-60.8)	60	0	13	17
	**2**	76.9 (49.7-91.8)	85.7 (76.2-91.8)	95.7 (88-98.5)	47.6 (28.4-67.6)	66	3	10	11
	**3**	100 (77.2-100)	66.2 (55.1-75.8)	100 (93-100)	33.3 (20.6-49)	51	0	13	26
	**4**	100 (77.2-100)	59.7 (48.6-70)	100 (92.3-100)	29.5 (18.2-44.2)	46	0	13	31
≥27	**1**	100 (77.2-100)	18.2 (11.2-28.2)	100 (78.5-100)	17.1 (10.3-27.1)	14	0	13	63
	**2**	100 (77.2-100)	28.6 (19.7-39.5)	100 (85.1-100)	19.1 (11.5-30)	22	0	13	55
	**3**	100 (77.2-100)	9.1 (4.5-17.6)	100 (64.6-100)	15.7 (9.4-25)	7	0	13	70
	**4**	100 (77.2-100)	0 (0-4.8)	NA	14.4 (8.6-23.2)	0	0	13	77

*(TP) and true negatives (TN). CI 95%; Confidence Interval 95%, PPV; Positive Predictive Value, NPV; Negative Predictive Value, FP; False Positive, TN; True Negative, FN; False Negative*

The table shows specificity, sensitivity, positive predictive value and negative predictive value of the non-standardized qualitative assessment and the semi-quantitative score

Rater 1 had one false positive (Supplementary Fig. 1) and rater had 2 two false positive predictions of poor outcome (Supplementary Fig. 2), none of the false-positives were mutual. Upon re-assessment five months later, Rater 1 did not have any false positives. The remaining two raters did not have any false positive predictions.

### Assessment by the CABI score

**Fig. 3:**
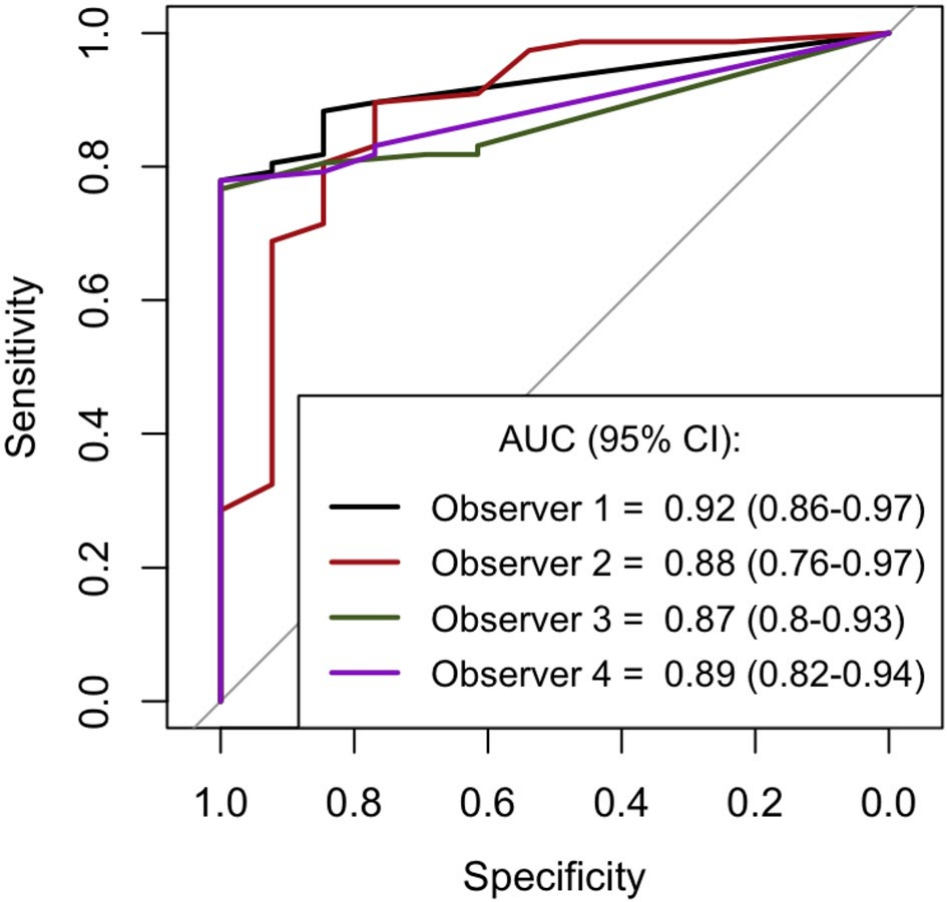
Receiver Operation Characteristics curve for prediction of functional outcome by the CABI score. The figure shows area under the receiver operating characteristics curve (AUC) with 95% confidence intervals for prediction of good versus poor outcome for each rater for the total sum of the CABI score

**Fig. 4:**
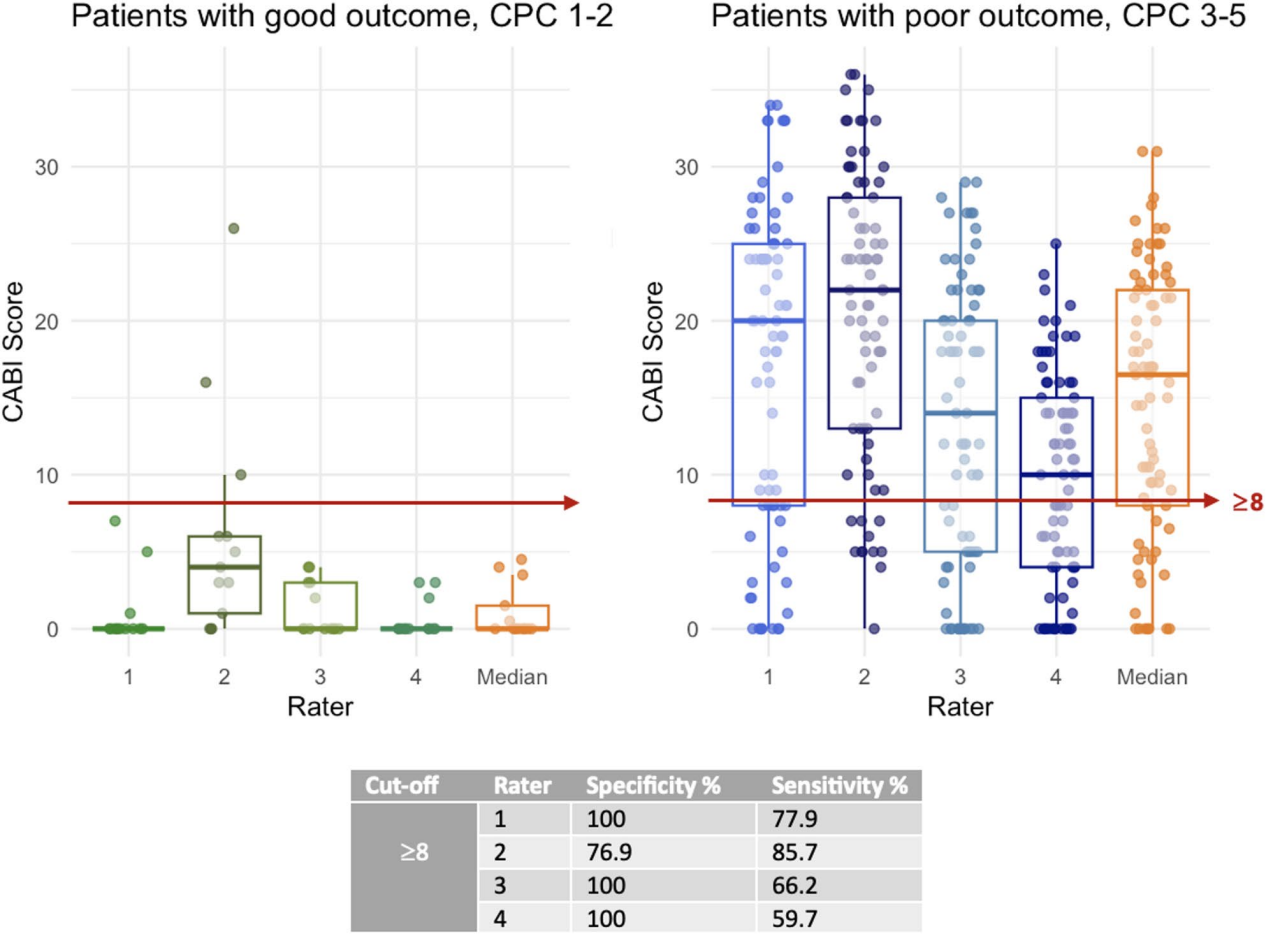
CABI score in patients with good and poor outcome. Boxplot with scatter for each of the four raters CABI scores in patients with good outcome (*n* = 13) versus poor outcome (*n* = 77), and the median score of all four raters, respectively. The specificity and sensitivity for poor outcome are presented at cut-off ≥8 (indicated by the red horizontal lines)

The CABI score (Fig. 1) predicted functional outcome with an area under the receiver operation curve of 0.87–0.92 for the four raters (Fig. 3), higher AUC than for the qualitative assessment (p-value: *p* = 0.02, 0.01, 0.03, 0.004 respectively). Figure 4 displays the distribution of the CABI score for good and poor outcome patients, respectively for all raters. The two outliers are the same as rater 2 false positives described above and in Supplementary Fig. 2. When evaluating cut-off scores for poor outcome prediction, the CABI score achieved a sensitivity of 59.7–85.7% and all but one rater reaching a specificity of 100% at cut-off ≥8 points (Table 2). The remaining rater demonstrated a specificity of 76.9%. The raters’ cut-offs yielding 100% specificity was ≥4, ≥5, ≥8 and ≥27 respectively. The corresponding sensitivities at these cut-offs ranged from 28.6 to 77.9%. Supplementary Fig. 3 presents Venn diagrams illustrating the overlap between cortical and deep brain involvement based on the CABI assessment by the four raters. The diagrams demonstrate that deep structure lesions rarely occur in isolation and are typically accompanied by cortical involvement.

### Inter- and intra-rater reliability

Inter-rater reliability for the qualitative assessment as per ERC/ESICM recommendations was moderate (Fleiss κ = 0.64, 95%CI: 0.53–0.75). Intra-rater reliability was high [Cohen’s κ = 0.9 (95%CI: 0.8–1)] for rater 1 who performed the qualitative assessment twice. The intraclass correlation coefficient (ICC) for the CABI score was 0.67 (95%CI: 0.58–0.75), reflecting moderate agreement among raters.

### Concordance between qualitative assessments and the CABI score

Table 3 compares the qualitative assessment as per ERC/ESICM recommendations with the results of the CABI scores assigned by each rater. Higher CABI scores generally aligned with the presence of extensive anoxic injury in qualitative assessments, whilst lower scores corresponded with the absence of extensive brain ischemia. Individual variability was noted; Raters 1 and 4 occasionally assigning low scores (2–4) in patients classified as with extensive anoxic injury, and Rater 3 assigning a high score of 26 in one patient classified without extensive anoxic injury. Despite these outliers, both the median with interquartile range (IQR) and the mean with 95% confidence intervals (CI) showed separation between the groups, suggesting that overall trends were consistent and not significantly affected by individual variability.

**Table 3:** Concordance between qualitative assessments and the CABI score

**Qualitative assessment**			**CABI score**		
**Rater**	Qualitative assessment ERC/ESICM criteria indicative of poor outcome	n (%)	min	max	median (IQR)
**1**	YES	60 (66.7)	2	34	23.5 (18-26)
	NO	30 (33.3)	0	10	0 (0-3)
**2**	YES	61 (67.8)	11	36	24 (20-29)
	NO	29 (32.2)	0	14	5 (4-9)
**3**	YES	47 (52.2)	10	29	20 (15.5-22.5)
	NO	43 (47.8)	0	26	1 (0-5)
**4**	YES	47 (52.2)	4	25	14 (11-18)
	NO	43 (47.8)	0	12	0 (0-4)

The table shows concordance between the qualitative radiological assessment per ERC/ESICM recommendations and the semi-quantitative CABI score. For example, rater 1 evaluated that 60 of 90 patients fulfilled ERC/ESICM criteria of a poor outcome and the remaining 30 patients did not. The median CABI score was 23.5 (IQR: 18-26) for patients fulfilling ERC/ESICM poor outcome criteria, compared to a median score 0 (IQR: 0-3) for patients not fulfilling poor outcome criteria on MRI

*CI 95%; Confidence Interval 95%, IQR; Interquartile range, min; Minimum, Max; Maximum, n; Number*

### Good outcome prediction

Only 13 patients in the study cohort had a good functional outcome, limiting statistical comparisons. A CABI score cut-off < 8 predicted good outcome with 76.9–100% sensitivity (Table 2; Figs. 3 and 4).

### Sensitivity analysis of the CABI score

We performed a predefined sensitivity analysis where the deep structures of MCA and PCA territories were excluded. This score predicted functional outcome with an area under the receiver operating characteristic curve (AUC) of 0.86–0.9 (Supplementary Fig. 4), similar AUC as for the CABI score (p-value: *p* = 0.06, 0.7, 0.5, 0.09 respectively). The distribution of scores in the sensitivity analysis for each rater, separated by good and poor outcomes, is shown in Supplementary Fig. 5. When evaluating cut-off scores for poor outcome prediction, the sensitivity analysis achieved a sensitivity of 67.5–90.9% while maintaining high specificity (84.6–100%) at cut-off ≥5 points (Supplementary Table 1). At a cut-off of ≥ 8 points, the raters achieved a sensitivity of 54.5–80.5%, with all but one rater reaching a specificity of 100%; the remaining rater demonstrated a specificity of 84.6%. The intraclass correlation coefficient (ICC) for the sensitivity analysis was 0.68 (95% CI: 0.59–0.75), indicating moderate reliability. Supplementary Table 2 shows concordance between qualitative assessments and the sensitivity analysis.

## Discussion

In this retrospective multicenter study, we found that the novel semi-quantitative CABI score for MRI evaluation had higher prognostic performance for predicting poor outcome after cardiac arrest, compared to the qualitative assessment recommended by guidelines.

The interrater agreement was moderate for the qualitative assessment as well as for the CABI score. Both the qualitative assessment and the CABI score assessment had false positive ratings, indicating that there is still a medical need for further improvement of MRI evaluation after cardiac arrest.

Prognostic modalities for prediction of poor outcome to guide treatment decisions should ideally be highly specific, preferably combined with a high sensitivity to reduce the number of patients classified with an indeterminate prognosis and a high reproducibility to reduce the variability between raters. In this study, the qualitative assessments as per ERC/ESICM recommendations demonstrated high specificity and sensitivity in line with previous results, emphasizing that MRI is an important tool for prognostication with high accuracy [3, 19]. Our finding of few false positive patients underscores the need to integrate MRI evaluation in a multimodal prognostication paradigm.

Most of the MRI scoring systems previously studied are based on the original framework by Hirsch et al., evaluating 21 brain regions, including cortical grey matter, subcortical white matter, deep grey nuclei, cerebellum, and brainstem [5]. The cortex score (including frontal, parietal, temporal, occipital, insula and hippocampus) has been considered the most practical, recent studies showed good prognostic performance both for the simplified and original versions of the cortex score and the deep grey nuclei score (caudate, putamen, globus pallidus and thalamus) [6, 8, 9]. Another previously investigated MRI scoring system (the DWI ASPECTs) evaluated 35 brain regions, mainly categorized by hemisphere (right/left, with exception for the brainstem) with a maximum score of 35 points and demonstrated high prognostic performance [10].

Similar to DWI-ASPECTs, the CABI score focuses on dividing the brain into its three vascular territories (ACA, MCA, PCA) with additional assessment of the cerebellum and the brainstem [10]. Cerebellar ischemia is more common post-arrest compared to brainstem ischemia [20], but has not shown to be a strong predictor of outcome after cardiac arrest. The inclusion of them in MRI scores have been discussed and they are therefore proportionally less weighted in the CABI-score [5, 6, 8, 9].

In line with previous studies, we had few false positive prognostications of poor outcome by MRI [6, 21]. Retrospective review of the images of the false positives, indicated one case with multiple ischemic lesions that could be interpreted as diffuse and extensive DWI lesions (Supplementary Fig. 1). One possible explanation for this false poor outcome prediction is that DWI signal in the cerebral cortex may differ from that in subcortical tissue in the healthy brain and thus, widespread restricted cortical diffusion may be erroneously assumed in patients with no hypoxic-ischemic encephalopathy (HIE). The lack of a clear definition and cutoff for pathological versus physiological DWI differences contributes to the difficulty of applying MRI for prognostication. A possible improvement to our scoring system may be to add standardized quantitative ADC thresholds for the assessments [22], either by ROI measurements or by standardized window settings, however such ADC thresholds would likely differ between MRI-scanners and would therefore require extensive work for standardization.

Compared to previously developed brain MRI scoring systems for neuroprognostication, our novel semi-quantitative score demonstrated similar sensitivity at 100% specificity [5, 6, 8, 9]. The timing of MRI is relevant for prognostic accuracy, with increasing sensitivity for hypoxic-ischemic brain injury after 2–3 days post-arrest, which is in accordance with the guideline recommendations for MRI [1, 7].

The deep grey nuclei score has demonstrated usefulness at an earlier timepoint than the cortex score, which showed higher sensitivity on days 5–7 [8], including both may therefore enhance the robustness of the scoring system. In our cohort, MRI was performed at a median of day 5, which may explain the strong performance of our sensitivity analysis, which gives relatively large emphasis on cortical regions. In this study, deep structural lesions rarely occurred in isolation and were typically accompanied by cortical involvement - an observation further supported by the similar prognostic performance reflected in the AUC values. A simpler cortex-dominated scoring system might be sufficient for practical use, but requires confirmation in larger cohorts.

MRI is rarely the first-line prognostic tool, often being reserved for cases where there is diagnostic uncertainty or complicating factors on CT. This may have contributed to the small number of patients with favorable outcomes (*n* = 13) in our study. MRI with low CABI score could be useful to exclude extensive brain injury and help identify potentially good outcome patients as recommended by ILCOR [4], but this should be evaluated in larger populations.

Magnetic resonance imaging has traditionally been limited to larger hospitals and is not always easily accessible. Recent studies demonstrated the feasibility of using a portable low-field MRI device at the bedside in intensive care settings, allowing repeated point-of-care and may thereby improve the access to MRI for post-arrest imaging in the future [23, 24].

In the future, it is likely that artificial intelligence (AI) tools may assist in standardizing imaging interpretation, making it more accessible to bedside clinicians [25]. These advancements have the potential to reduce variability in assessments and improve prognostic accuracy. On the other hand, AI assisted MRI interpretation could be problematic, if false predictions due to unknown factors unrelated to brain injury are possible. Recent studies have explored the use of functional MRI (fMRI) for prognostication in unconscious patients in the ICU [26, 27], offering insights into brain activity and function that may complement traditional structural imaging in critical care settings. As recommended in the ERC/ESICM guidelines, we emphasize the importance of applying a multimodal approach to prognostication to reduce the risk of misclassifications within neurological prognostication after cardiac arrest [1].

### Strengths and limitations

A key strength of the study is that brain MRI assessments were performed by four radiology raters blinded to clinical data using a standardized checklist. Additionally, the cohort was reflective of real-life clinical practice, including both in-hospital cardiac arrest and out-of-hospital cardiac arrest patients. MRI was frequently performed in patients with suspected severe brain injury, causing a selection of poor outcome patients. As a result, our cohort included only 13 patients with good outcome and 95% confidence intervals on specificity are wide. Withdrawal of life-sustaining therapies was practiced and decisions included on local radiologists’ evaluation of MRI images in addition to other methods of neurological prognostication as per guideline recommendations. The risk of self-fulfilling prophecies cannot be excluded. Furthermore, selection bias may be present, as MRI was more often performed in patients with poor outcomes, potentially overestimating predictive performance. The CABI score may also overestimate hypoxic-ischemic brain injury severity, as all chemical hits were rated, potentially including small embolic lesions with limited prognostic significance. Despite using a standardized checklist for radiological evaluation, the radiologists’ qualitative assessments might have been influenced by parallel evaluation of the semi-quantitative score.

## Conclusion

A standardized approach to MRI evaluations after cardiac arrest could improve prognostic reliability compared to qualitative assessments recommended by guidelines. Both radiological assessment methods demonstrated moderate inter-rater agreement and individual false positive ratings occurred, indicating the need of additional standardization. Our results should be prospectively validated ideally in cohorts in which withdrawal of life-sustaining therapies is not practiced.

## Supplementary Information

Supplementary Material 1.

Supplementary Material 2.

Supplementary Material 3.

## Data Availability

The protocol and the datasets analysed during the current study are available from the corresponding author upon reasonable request.

## Abbreviations

AUC: Area under the receiver operation curve
CI: Confidence interval
CPC: Cerebral performance category
DGN: deep grey nuclei
DWI: Diffusion-Weighted Imaging
FLAIR: Fluid-Attenuated Inversion Recovery
FN: False negative
FP: False positive
ICU: Intensive care unit
IHCA: In-hospital cardiac arrest
IQR: Interquartile range
MRI: Magnetic Resonance Imaging
NPV: Negative predictive value
OHCA: Out-of-hospital cardiac arrest
PPV: Positive predictive value
TP: True positive
TN: True negative
WLST: Withdrawal of life-sustaining therapy
