# Supplementary material for: The semi-quantitative cardiac arrest brain ischemia (CABI) score for magnetic resonance imaging predicts functional outcome after cardiac arrest
Source: Crit Care. 2025 Aug 20;29:373. doi: 10.1186/s13054-025-05595-1 (PMC12369042; doi:10.1186/s13054-025-05595-1)
Supplement: Supplementary file 1 — Supplementary Material 1. [file 13054_2025_5595_MOESM1_ESM.docx]

**Supplementary Material**

The semi-quantitative Cardiac Arrest Brain Ischemia (CABI) score for magnetic resonance imaging predicts functional outcome after cardiac arrest

Isabelle Arctaedius^1^, Johan Wassélius^2^, Margareta Lang^3^, Mattias Drake^2^, Mikael Johnsson^4^, Hans Friberg^5^, Christoph Leithner^6^, Martin Kenda^6^, Anna Lybeck^1^ and Marion Moseby-Knappe^7^

1. Lund University, Skåne University Hospital, Department of Clinical Sciences, Anaesthesia & Intensive Care, Lund, Sweden
2. Lund University, Skåne University Hospital, Department of Clinical Sciences, Medical Imaging and Physiology, Lund, Sweden
3. Lund University, Helsingborg Hospital, Department of Clinical Sciences, Radiology, Helsingborg, Sweden.
4. Helsingborg Hospital, Department of Radiology, Helsingborg, Sweden
5. Lund University, Skåne University Hospital, Department of Clinical Sciences, Anaesthesia & Intensive Care, Malmö, Sweden
6. Charité – Universitätsmedizin Berlin, corporate member of Freie Universität Berlin and Humboldt-Universität zu Berlin, Department of Neurology and Experimental Neurology, Berlin, Germany
7. Lund University, Skåne University Hospital, Department of Clinical Sciences, Neurology and Rehabilitation Medicine, Lund, Sweden.

*Corresponding author:*

Isabelle Arctaedius (isabelle.arctaedius@med.lu.se)

Telephone: +46 (0)46 17 42 33

Department of Anaesthesia & Intensive Care

Skåne University Hospital

Entrégatan 7

SE-222 42 Lund Sweden
Orchid-ID: 0000-0001-7857-7393

## Table of content

1. Supplementary table 1. Specificity, sensitivity and predictive values of the qualitative assessment and the sensitivity analysis 4

2. Supplementary table 2. Concordance between qualitative assessments and the sensitivity analysis5

3. Supplementary figure 1. MRI brain images of false positive evaluations6

4. Supplementary figure 2. MRI brain images of outliers7

5. Supplementary figure 3. Overlap between cortical and deep brain lesions by hemisphere and vascular territory 8

6. Supplementary figure 4. Receiver Operation Characteristics curve for prediction of functional outcome by the sensitivity analysis9

7. Supplementary figure 5. The sensitivity analysis in patients with good and poor outcome10

## Supplementary Table 1. Specificity, sensitivity and predictive values of the qualitative assessment and the sensitivity analysis

| Qualitative assessment per ERC/ESICM recommendations | | | | | | | | | |
| --- | --- | --- | --- | --- | --- | --- | --- | --- | --- |
|  | Rater | Specificity % (95%CI) | Sensitivity % (95%CI) | PPV % (95%CI) | NPV % (95%CI) | TP | FP | TN | FN |
|  | 1 | 92.3 (66.7-98.6) | 76.6 (66-84.7) | 98.3 (91.1-99.7) | 40 (24.6-57.7) | 59 | 1 | 12 | 18 |
|  | 2 | 84.6 (57.8-95.7) | 76.6 (66-84.7) | 96.7 (88.8-99.1) | 37.9 (22.7-56) | 59 | 2 | 11 | 18 |
|  | 3 | 100 (77.2-100) | 61 (49.9-71.2) | 100 (92.4-100) | 30.2 (18.6-45.1) | 47 | 0 | 13 | 30 |
|  | 4 | 100 (77.2-100) | 61 (49.9-71.2) | 100 (92.4-100) | 30.2 (18.6-45.1) | 47 | 0 | 13 | 30 |
| Sensitivity analysis | | | | | | | | | |
| Score (0-24p) | Rater | Specificity % (95%CI) | Sensitivity % (95%CI) | PPV % (95%CI) | NPV % (95%CI) | TP | FP | TN | FN |
| ≥4 | 1 | 84.6 (57.8-95.7) | 79.2 (68.9-86.8) | 96.8 (89.1-99.1) | 40.7 (24.5-59.3) | 61 | 2 | 11 | 16 |
|  | 2 | 69.2 (42.4-87.3) | 92.2 (84-96.4) | 94.7 (87.1-97.9) | 60 (35.7-80.2) | 71 | 4 | 9 | 6 |
|  | 3 | 84.6 (57.8-95.7) | 75.3 (64.6-83.6) | 96.7 (88.6-99.1) | 36.7 (21.9-54.5) | 58 | 2 | 11 | 19 |
|  | 4 | 100 (77.2-100) | 72.7 (61.9-81.4) | 100 (93.6-100) | 38.2 (23.9-55) | 56 | 0 | 13 | 21 |
| ≥5 | 1 | 92.3 (66.7-98.6) | 76.6 (66-84.7) | 98.3 (91.1-99.7) | 40 (24.6-57.7) | 59 | 1 | 12 | 18 |
|  | 2 | 84.6 (57.8-95.7) | 90.9 (82.4-95.5) | 97.2 (90.4-99.2) | 61.1 (38.6-79.7) | 70 | 2 | 11 | 7 |
|  | 3 | 100 (77.2-100) | 74 (63.3-82.5) | 100 (93.7-100) | 39.4 (24.7-56-3) | 57 | 0 | 13 | 20 |
|  | 4 | 100 (77.2-100) | 67.5 (56.5-76.9) | 100 (93.1-100) | 34.2 (21.2-50.1) | 52 | 0 | 13 | 25 |
| ≥8 | 1 | 100 (77.2-100) | 71.4 (60.5-80.3) | 100 (93.5-100) | 37.1 (23.2-53.7) | 55 | 0 | 13 | 22 |
|  | 2 | 84.6 (57.8-95.7) | 80.5 (70.3-87.8) | 96.9 (89.3-99.1) | 42.3(25.5-61.1) | 62 | 2 | 11 | 15 |
|  | 3 | 100 (77.2-100) | 63.6 (52.5-73.5) | 100 (92.7-100) | 31.7 (19.6-47) | 49 | 0 | 13 | 28 |
|  | 4 | 100 (77.2-100) | 54.5 (43.5-65.2) | 100 (91.6-100) | 27.1 (16.6-41) | 42 | 0 | 13 | 35 |
| ≥17 | 1 | 100 (77.2-100) | 40.3 (30-51.4) | 100 (89-100) | 22 (13.4-34.1) | 31 | 0 | 13 | 46 |
|  | 2 | 100 (77.2-100) | 40.3 (30-51.4) | 100 (89-100) | 22 (13.4-34.1) | 31 | 0 | 13 | 46 |
|  | 3 | 100 (77.2-100) | 22.1 (14.3-32.5) | 100 (81.6-100) | 17.8 (10.7-28.1) | 17 | 0 | 13 | 60 |
|  | 4 | 100 (77.2-100) | 9.1 (4.5-17.6) | 100 (64.6-100) | 15.7 (9.4-25) | 7 | 0 | 13 | 70 |

The table shows specificity, sensitivity, positive predictive value and negative predictive value of the sensitivity analysis and the raters cut-off yielding 100% specificity. The sensitivity analysis includes the regions of the CABI score in addition to MCA deep and PCA deep bilaterally. (TP) and true negatives (TN). CI 95%; Confidence Interval 95%, PPV; Positive Predictive Value, NPV; Negative Predictive Value, FP; False Positive, TN; True Negative, FN; False Negative.

## Supplementary table 2. Concordance between qualitative assessments and the sensitivity analysis

|  | Qualitative assessment | | Sensitivity analysis | | |
| --- | --- | --- | --- | --- | --- |
| Rater | Qualitative assessment ERC/ESICM criteria indicative of poor outcome | n (%) | min | max | median (IQR) |
| 1 | YES | 60 (66.7) | 2 | 22 | 17 (12 - 18) |
|  | NO | 30 (33.3) | 0 | 9 | 0 (0 - 1.8) |
| 2 | YES | 61 (67.8) | 8 | 24 | 17 (14 - 20) |
|  | NO | 29 (32.2) | 0 | 10 | 3 (1 - 6) |
| 3 | YES | 47 (52.2) | 8 | 21 | 15 (12 - 18) |
|  | NO | 43 (47.8) | 0 | 18 | 0 (0 - 4) |
| 4 | YES | 47 (52.2) | 1 | 21 | 12 (9 - 15) |
|  | NO | 43 (47.8) | 0 | 10 | 0 (0 – 3.5) |

The table shows concordance between the qualitative radiological assessment per ERC/ESICM recommendations and the sensitivity analysis. For example, rater 1 evaluated that 60 of 90 patients fulfilled ERC/ESICM criteria of a poor outcome and the remaining 30 patients did not. The median score was 17 (IQR: 12-18) for patients fulfilling ERC/ESICM poor outcome criteria, compared to a median score 0 (IQR: 0-1.8) for patients not fulfilling poor outcome criteria on MRI.

*CI 95%; Confidence Interval 95%, IQR; Interquartile range, min; Minimum, Max; Maximum, n; Number.*

## Supplementary Figure 1. MRI brain images of false positive evaluations

**
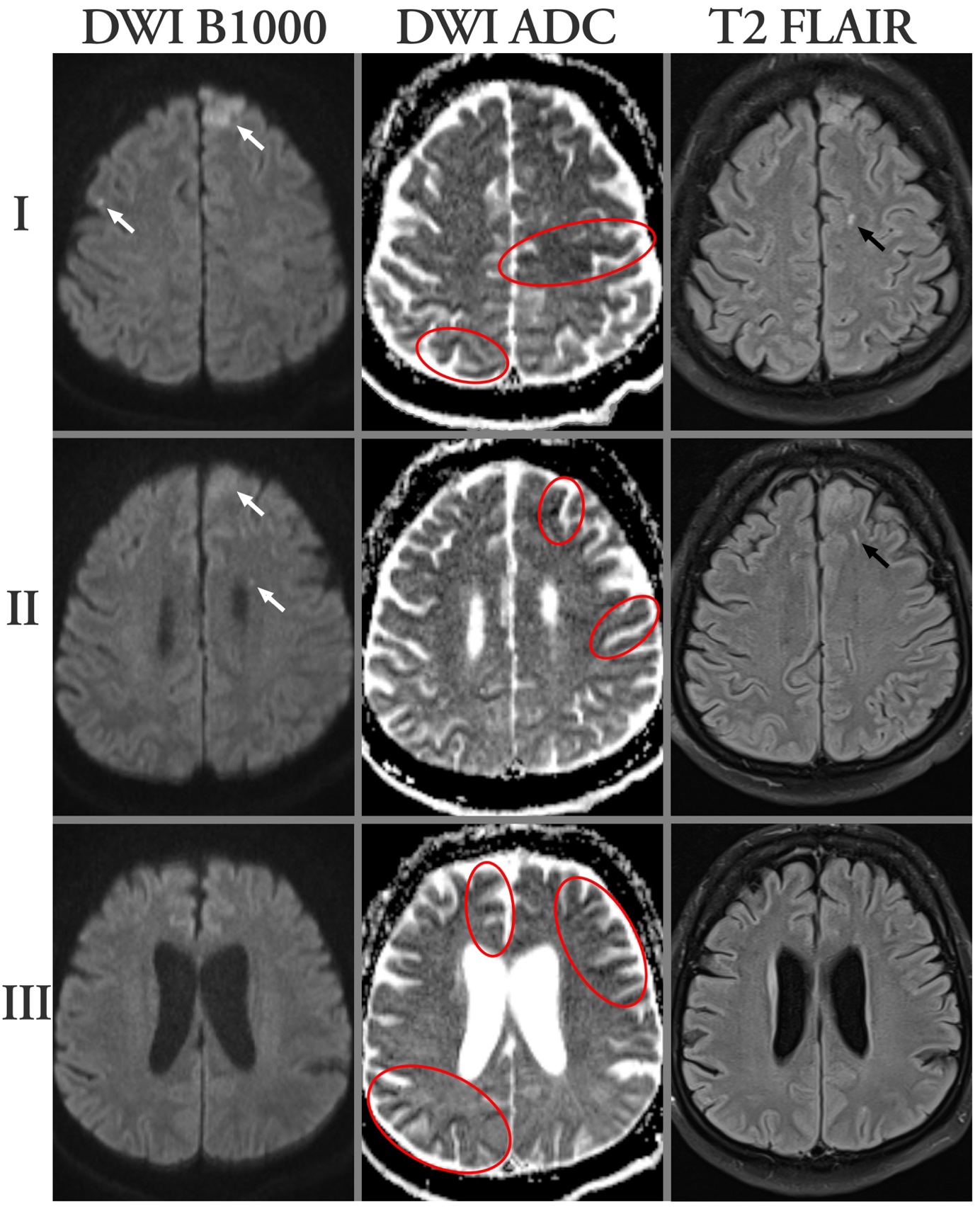
**

The figures show DWI B1000 and ADC and T2 FLAIR images from a false positive case at three supratentorial levels (I, II and III) showing three small ischemic lesions on B1000 (white arrows), larger areas of restricted diffusion on ADC (Red ellipsoids) and two hyperdense white matter lesions on T2 FLAIR (black arrows), presumed to be old non-specific lesions. Based on lesions on B1000 the patient would get a score of 3. Including the suspected diffusion abnormalities on ADC, the score would be at least 6. The four raters scored 7, 6, 3 respectively 2 on the CABI score.

## Supplementary Figure 2. MRI brain images of outliers
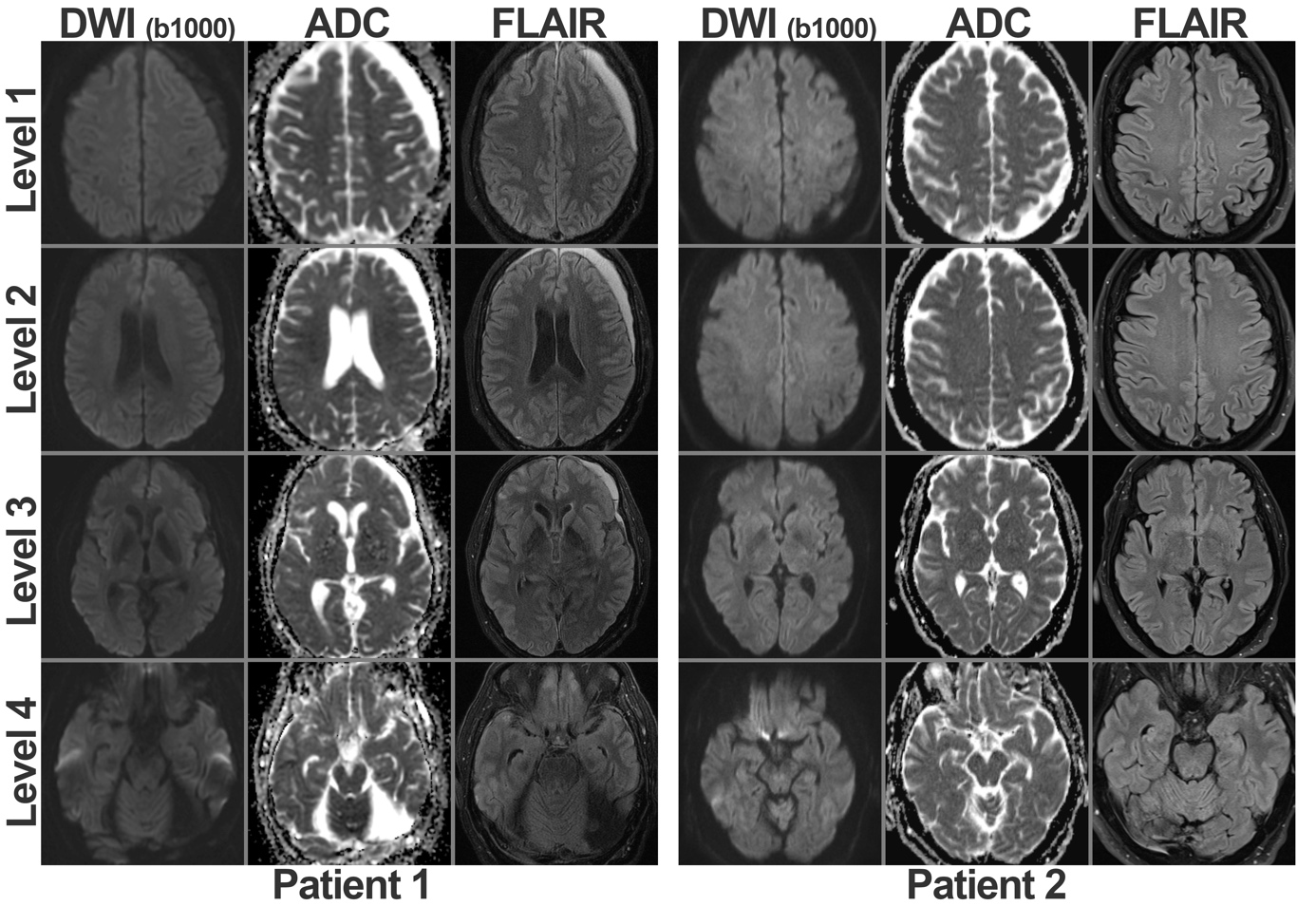


Image examples from two outlier cases rated substantially higher by one compared to the other raters. DWI (b1000), ADC and FLAIR images at four transaxial levels are shown.

Supplementary Figure 3. Overlap between cortical and deep brain lesions by hemisphere and vascular territory


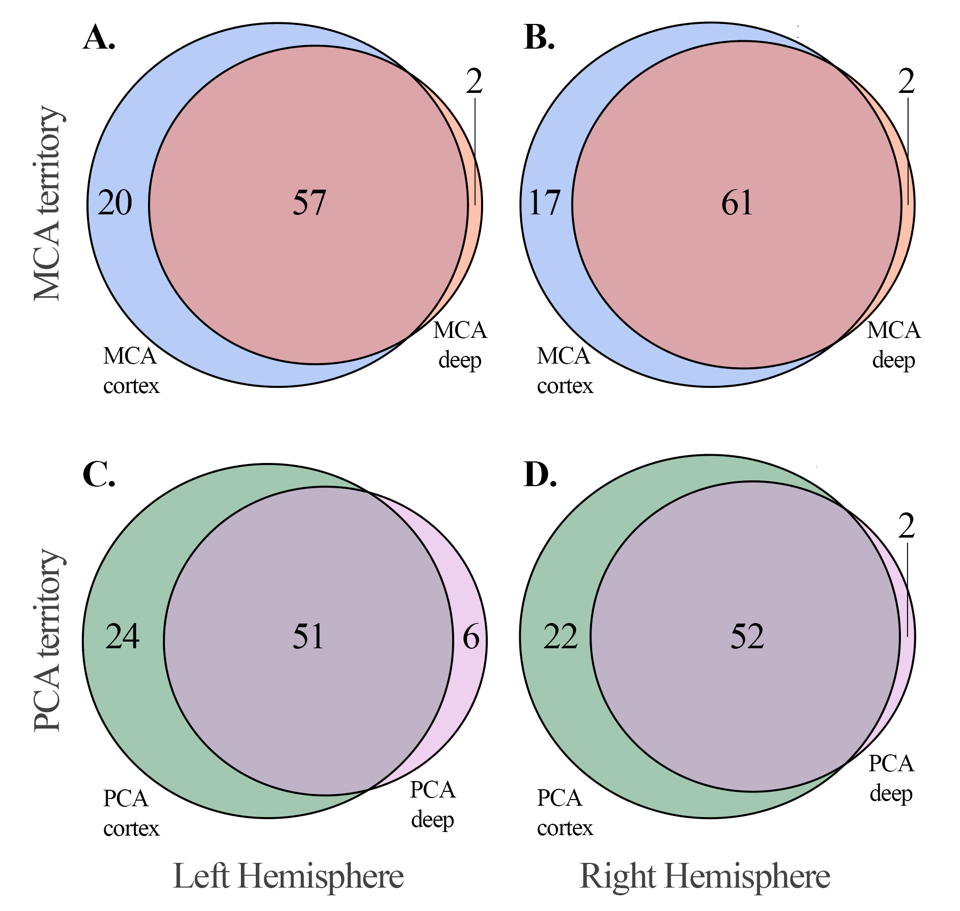


Venn diagrams (A–D) display the overlap between cortical and deep involvement based on visual assessment by the four raters. For each patient, any region with a score > 0 from at least one rater was classified as involved. Patients with no involvement in either region (i.e., score = 0 across all raters) were excluded from the diagrams.

(A) Left hemisphere: MCA cortex vs MCA deep (basal ganglia)

(B) Right hemisphere: MCA cortex vs MCA deep (basal ganglia)

(C) Left hemisphere: PCA cortex vs PCA deep (thalamus)

(D) Right hemisphere: PCA cortex vs PCA deep (thalamus)

The diagrams demonstrate the distribution and co-occurrence of injury across these anatomical regions

## Supplementary Figure 4. Receiver Operation Characteristics curve for prediction of functional outcome by the sensitivity analysis

##
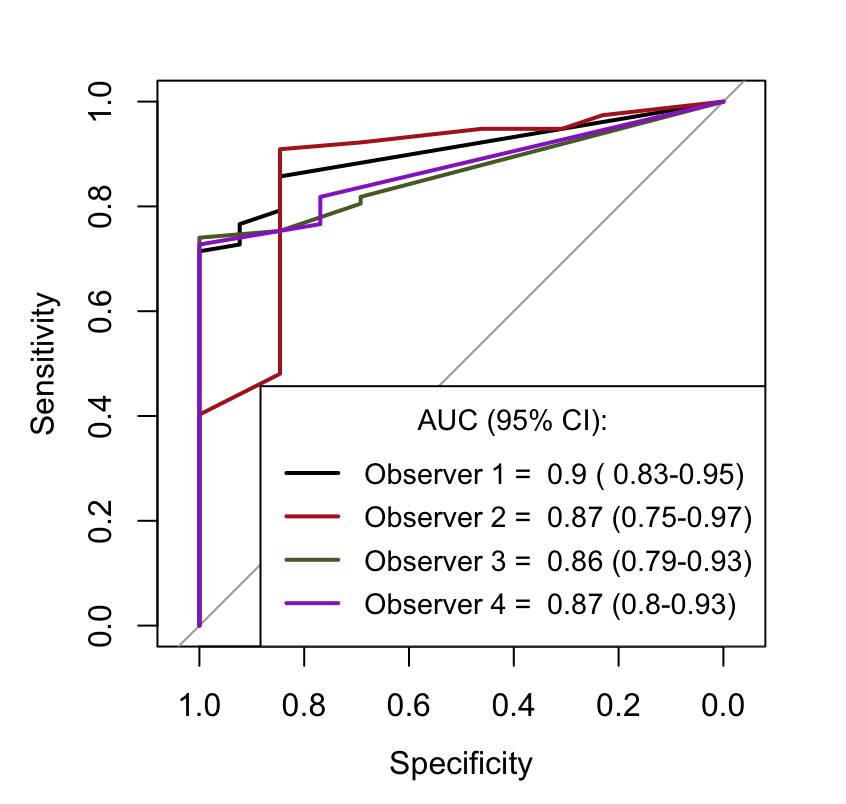


The figure shows Area Under the Receiver Operation Characteristics Curve (AUC) with 95% Confidence intervals for prediction of good versus poor outcome for each rater for the sensitivity analysis. The sensitivity analysis includes the regions of the CABI score minus the MCA deep and PCA deep bilaterally.

## Supplementary Figure 5. The sensitivity analysis in patients with good and poor outcome
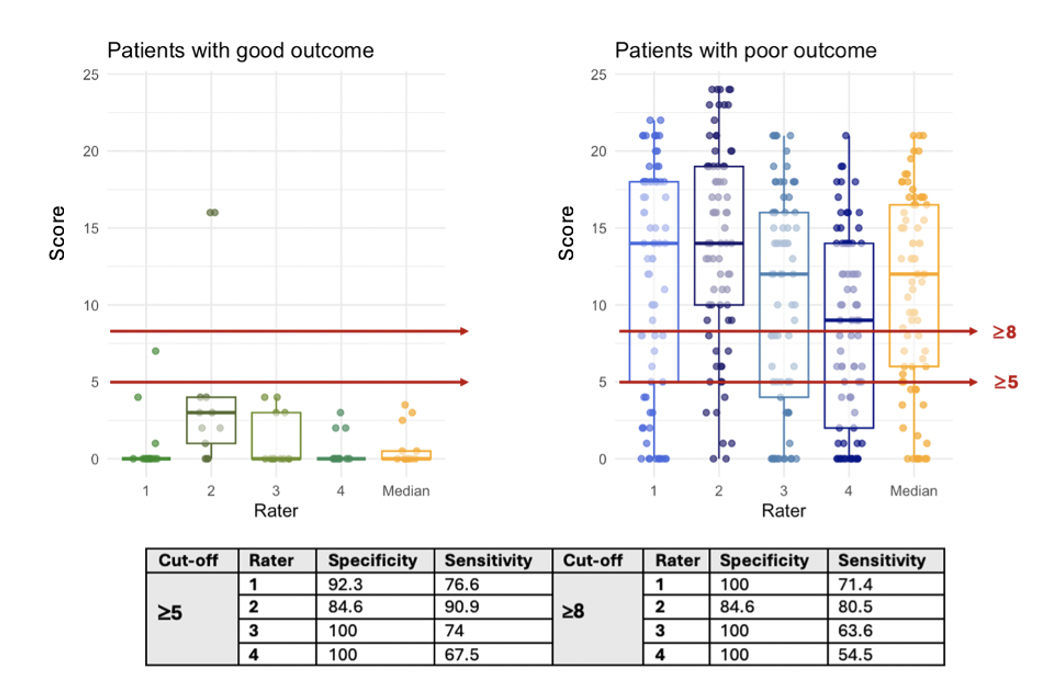


Boxplot with scatter for each of the four raters scores in the sensitivity analysis for patients with good outcome (n=13) versus poor outcome (n=77), and the median score of all four raters, respectively. The sensitivity analysis includes the regions of the CABI score minus MCA deep and PCA deep bilaterally. The specificity and sensitivity for poor outcome are presented at cut-offs ≥5 and ≥8 (indicated by the red horizontal lines).
