# Supplementary material for: The semi-quantitative cardiac arrest brain ischemia (CABI) score for magnetic resonance imaging predicts functional outcome after cardiac arrest
Source: Crit Care. 2025 Aug 20;29:373. doi: 10.1186/s13054-025-05595-1 (PMC12369042; doi:10.1186/s13054-025-05595-1)
Supplement: Supplementary file 2 — Supplementary Material 2. [file 13054_2025_5595_MOESM2_ESM.pptx]

## Slide 1
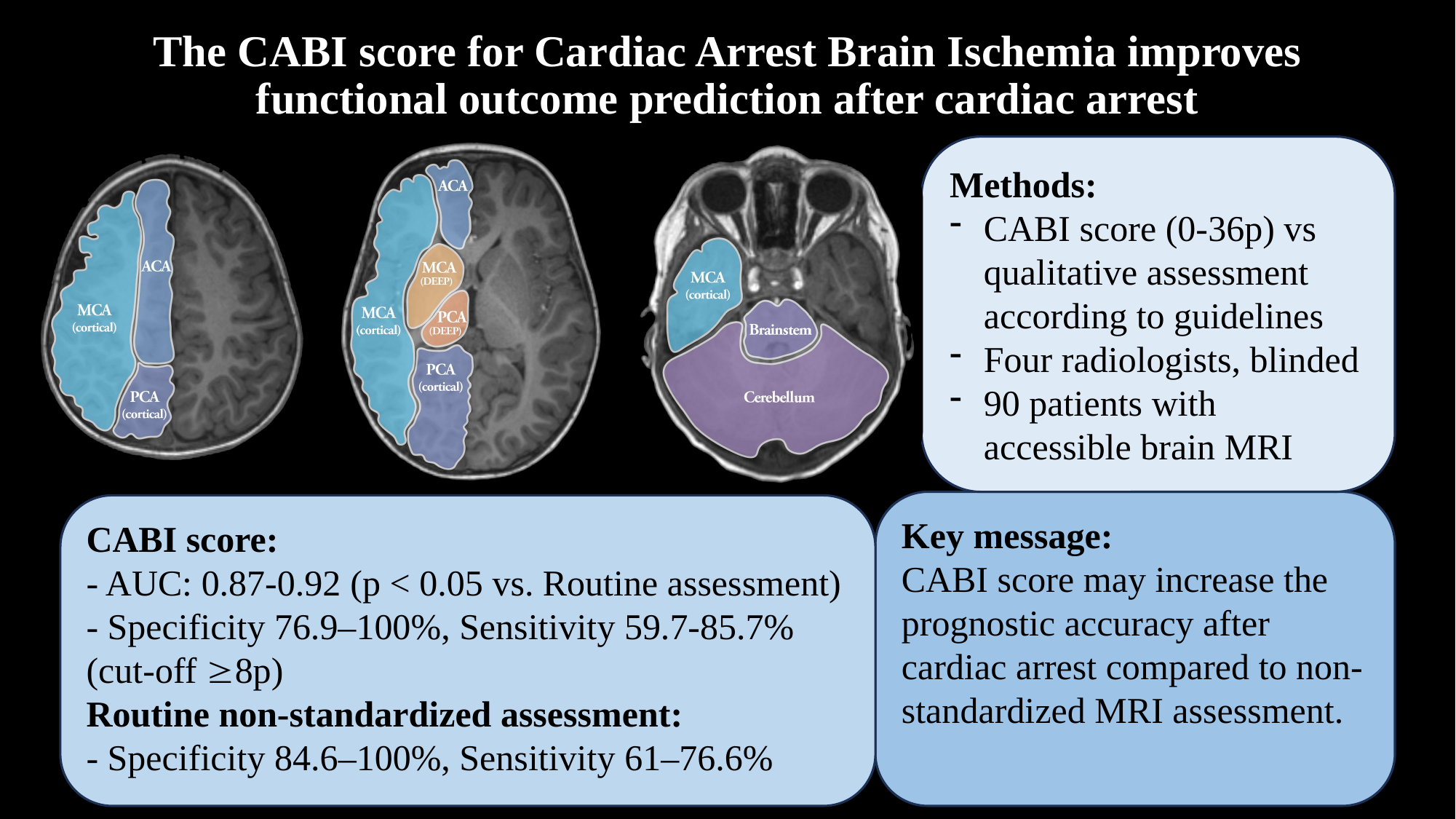

# The CABI score for Cardiac Arrest Brain Ischemia improves functional outcome prediction after cardiac arrest
Methods:
CABI score (0-36p) vs qualitative assessment according to guidelines
Four radiologists, blinded
90 patients with accessible brain MRI
Key message:
CABI score may increase the prognostic accuracy after cardiac arrest compared to non-standardized MRI assessment.
CABI score:
- AUC: 0.87-0.92 (p < 0.05 vs. Routine assessment)
- Specificity 76.9–100%, Sensitivity 59.7-85.7% (cut-off ≥8p)
Routine non-standardized assessment:
- Specificity 84.6–100%, Sensitivity 61–76.6%
