# Supplementary figures and images for: The semi-quantitative cardiac arrest brain ischemia (CABI) score for magnetic resonance imaging predicts functional outcome after cardiac arrest
Source: Crit Care. 2025 Aug 20;29:373. doi: 10.1186/s13054-025-05595-1 (PMC12369042; doi:10.1186/s13054-025-05595-1)

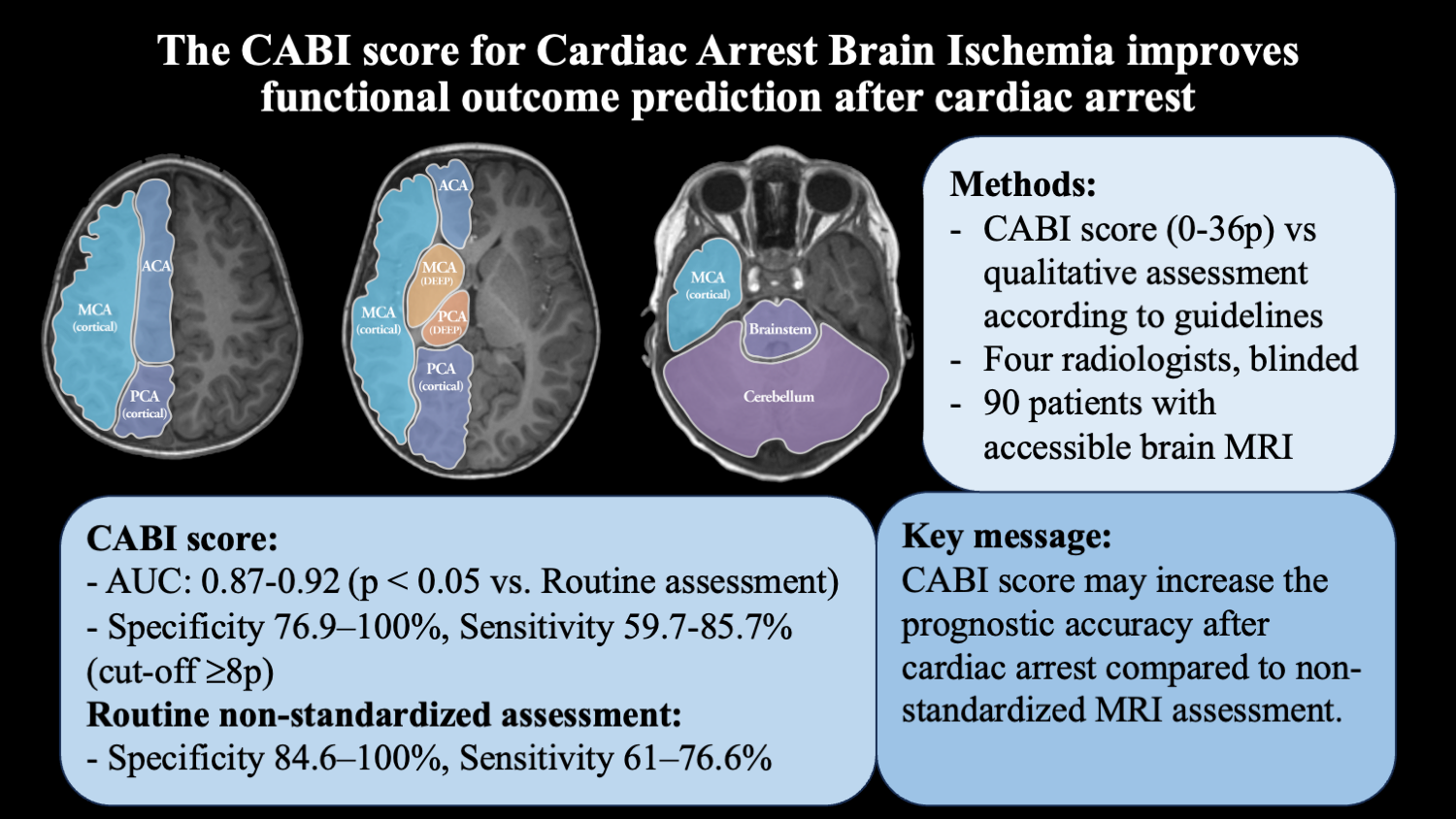

Supplement: Supplementary file 3 — Supplementary Material 3. [file 13054_2025_5595_MOESM3_ESM.tiff]
